# A Rare Collision Tumor: Adenocarcinoma in the Ampulla of Vater and Neuroendocrine Tumor in the Lower Part of the Common Bile Duct

**DOI:** 10.7759/cureus.15882

**Published:** 2021-06-24

**Authors:** Mitsuhiro Tachibana, Kazuyasu Kamimura, Kei Tsukamoto, Yutaka Tsutsumi

**Affiliations:** 1 Department of Diagnostic Pathology, Shimada City General Medical Center, Shimada, JPN; 2 Department of Surgery, Shimada City General Medical Center, Shimada, JPN; 3 Department of Diagnostic Radiology, Shimada City General Medical Center, Shimada, JPN; 4 Diagnostic Pathology Clinic, Pathos Tsutsumi, Inazawa, JPN

**Keywords:** collision tumor, ampulla of vater, common bile duct, adenocarcinoma, well differentiated neuroendocrine tumor

## Abstract

In the biliary tree, only three cases of neuroendocrine tumor (NET) synchronous with adenocarcinoma have been reported so far. We experienced a case of NET, grade 2 (G2), of the common bile duct associated with adenocarcinoma of the ampulla of Vater (AoV). A Japanese man at his 60’s visited a local doctor, and obstructive jaundice was pointed out. Under the clinical diagnosis of carcinoma of the AoV, 20 x 20 mm, T3aN0M0 stage IIB, pylorus-preserving pancreaticoduodenectomy was performed. Dynamic computed tomography in an artery-dominant phase and microscopic examination revealed that the mass consisted of two different components; hypovascular, 2.5 cm-sized, exophytic adenocarcinoma in the AoV and hypervascular, 1.5 cm-sized, polypoid NET (G2) in the lower part of the common bile duct. The NET diffusely expressed neuroendocrine markers, including somatostatin receptor-2 (SSTR2), and adenocarcinoma cells arising from tubular adenoma focally expressed the neuroendocrine markers. Both malignancies were positive for caudal-type homeobox-2 (CDX2). It is presumed that NET occurred from intestinal-type adenoma/adenocarcinoma.

## Introduction

Neuroendocrine tumors (NETs) account for 0.5% of all the newly diagnosed malignancies [1]. NETs show a histopathological spectrum encompassing NET G1 to G3 and neuroendocrine carcinoma (small cell type or large cell type). Most of these tumors are non-functioning while they occasionally secrete serotonin, accompanying symptoms of flushing, diarrhea and hypotension. The carcinoid syndrome is seen predominantly in tumors of the ileum and vermiform appendix of mid-gut origin and the lung [2].

We report herein a case of NET of the biliary tree, concordant with adenocarcinoma component. No hormonal symptoms were associated. To the best of our knowledge, only three cases of a collision tumor of the bile duct system, consisting of NET and adenocarcinoma, have been reported in the literature [3-5].

## Case presentation

Clinical summary

**Figure 1:**
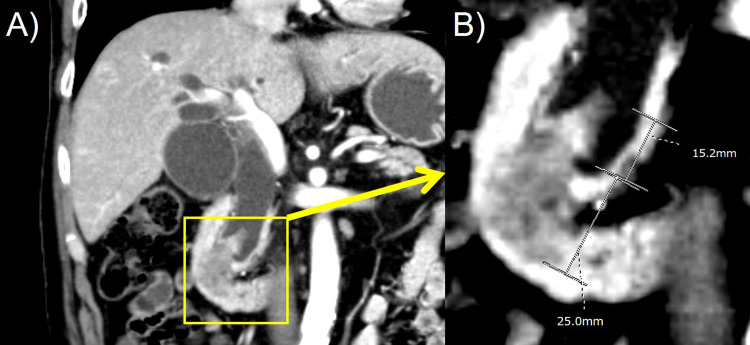
Radiological findings of tumors of the ampulla of Vater (AoV) and common bile duct (CBD) (A,B). The dynamic CT study in an artery-dominant phase indicates that the mass consists of two components: a hypovascular, 2.5 cm-sized exophytic tumor component in the AoV and a hypervascular, 1.5 cm-sized polypoid tumor component in the lower part of the CBD.

A Japanese man at his 60’s visited a local doctor with a complaint of malaise, and obstructive jaundice was pointed out. Based on the persistence of malaise, he had stopped smoking just one month before. No family history of neoplasm was recorded. Physical examination revealed no tumor mass or tenderness on the abdomen. Laboratory tests indicated hyperbilirubinemia (13.1 mg/dL) and an elevated level of carbohydrate antigen 19-9 (43.6 U/mL; range 0-33). Abdominal ultrasonography demonstrated the dilatation of the bile duct. The patient underwent endoscopic retrograde cholangiopancreatography: the ampulla of Vater (AoV) was markedly swollen and covered with smooth-surfaced mucosa. A polypoid mass was seen in the lower part of the common bile duct (CBD). Dynamic computed tomography (CT) in an artery-dominant phase indicated that the mass consisted of two components: a hypovascular, 2.5 cm-sized, exophytic tumor of the AoV and a hypervascular, 1.5 cm-sized polypoid tumor of the CBD (Figure 1).

Under the diagnosis of carcinoma of the AoV, 20 x 20 mm, T3aN0M0 Stage IIB, pylorus-preserving pancreaticoduodenectomy was performed. Adjuvant chemotherapy with TS1 was discontinued because of the complication of anorexia, stomatitis and rash on the skin. He remained well without recurrence or metastasis 13 months after surgery.

Macroscopic, light microscopic, and immunohistochemical analysis of surgical material

**Figure 2:**
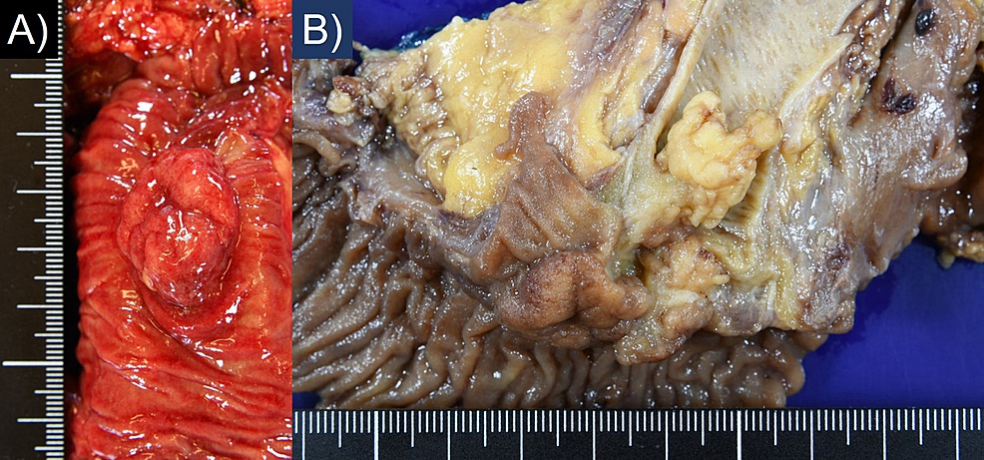
Macroscopic findings of the tumors of the ampulla of Vater (AoV) and the lower part of the common bile duct (CBD). (A) The AoV mass is an exophytic tumor measuring 26 x 22 mm. (B) The polypoid tumor of the CBD measuring 15 x 12 mm is seen just adjacent to the polypoid tumor of the AoV.

**Figure 3:**
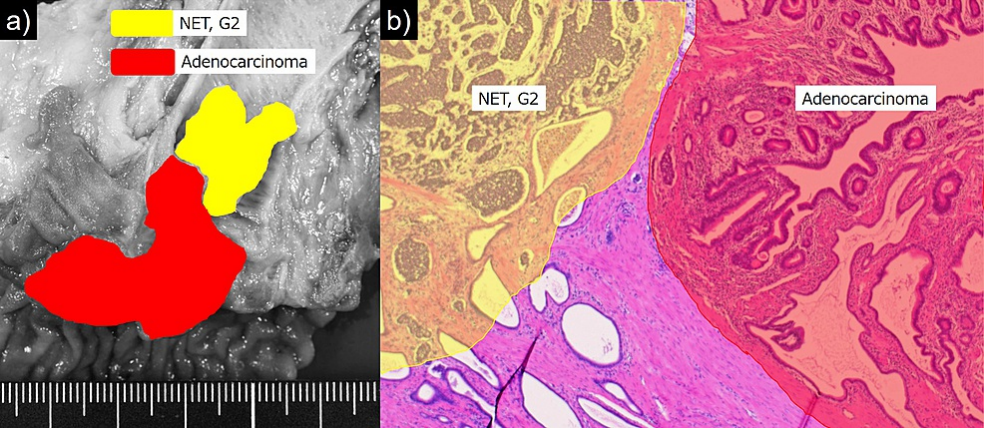
Comparative and schematic presentation of the present neoplasm. The hypovascular ampulla of Vater (AoV) tumor belongs to adenocarcinoma arising from tubulovillous adenoma (red area), while the hypervascular common bile duct (CBD) mass represents neuroendocrine tumor (NET), G2 (yellow area).

Grossly, the surgical material contained two neoplastic components: an exophytic tumor of the AoV measuring 26 x 22 mm and a 15 x 12 mm-sized polypoid tumor of the lower part of the CBD, as illustrated in Figure 2 and Figure 3.

Both tumors collided at the lowest part of the CBD. Microscopically, the CBD tumor was diagnosed as NET, G2 with Ki-67 labeling index of 3%. The tumor cells expressed such neuroendocrine markers as chromogranin A, synaptophysin, CD56 (neuronal cell adhesion molecule: NCAM) and somatostatin receptor-2 (SSTR2). CDX2 was also positive. Lymphovascular invasion was detected.

**Figure 4:**
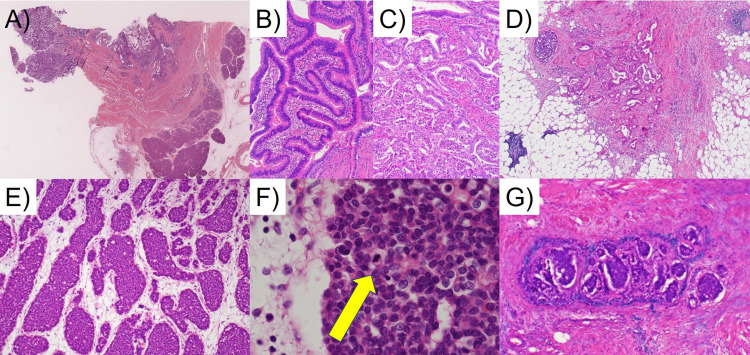
Light microscopic findings of the tumors of the ampulla of Vater (AoV) and common bile duct (CBD). (A) A low-powered view of the lower CBD indicates collision tumors. Invasive adenocarcinoma is noted on the right side, while neuroendocrine tumor (NET) is observed on the left side (H&E). (B) Tubulovillous adenoma of the AoV (H&E). (C) Well to moderately differentiated adenocarcinoma of the AoV (H&E). (D) The invasion front of adenocarcinoma in the subserosal layer (H&E). (E) NET, G2 in the lower part of the CBD (H&E). (F) High power view of NET, G2 demonstrates occasional mitotic activity (arrow) (H&E). (G) Vascular invasion of NET, G2 (Victoria blue H&E).

**Figure 5:**
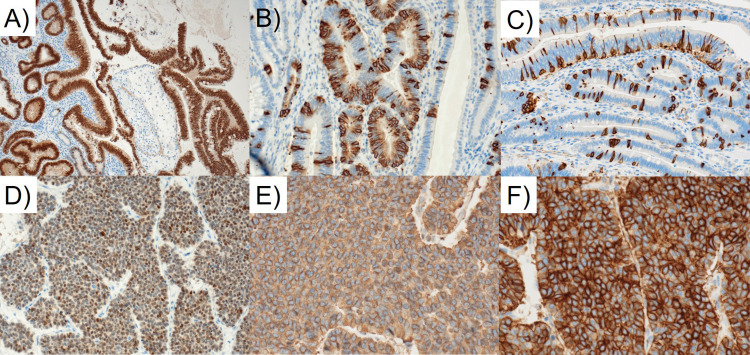
Immunohistochemical findings of the tumors of the ampulla of Vater (AoV) (A-C) and common bile duct (CBD) (D-F). Adenocarcinoma of the AoV expresses CDX2 diffusely (A), and chromogranin A (B) and SSTR-2 (C) occasionally. Neuroendocrine tumor (NET), G2, of the common bile duct is diffusely immunoreactive for CDX2 (D), chromogranin A (E) and SSTR-2 (F).

The second exophytic neoplasm of the AoV was well-differentiated adenocarcinoma arising from tubular adenoma and invading the subserosa with lymphatic permeation. The cancer cells occupied 70% of the neoplasm of AoV. Both the cancer cells and adenoma cells were immunoreactive for CDX2, mucin 2 (MUC2), MUC5AC, and MUC6. Chromogranin A, synaptophysin and SSTR2 were focally positive. Representative histological and immunohistochemical findings are illustrated in Figure 4 and Figure 5, respectively.

One of 33 lymph nodes evaluated was positive for metastasis of both adenocarcinoma and NET. The final pathological stages, based on the American Joint Committee on Cancer and the International Union Against Cancer (UICC) staging system (the 8th edition), were pT3aN1Mx G1 for ampullary adenocarcinoma and pT2N1Mx G2 for NET of the CBD.

## Discussion

NETs of the gastroenteropancreatic (GEP) system originate from the diffuse neuroendocrine (NE) system encompassing 15 types of differentiated NE cells located in the gastrointestinal tract and pancreas. The most common location of GEP/NETs in Caucasian people is the vermiform appendix (62%), and they may be seen in the pancreas (2%), small intestine (27%), undefined primary locations with hepatic metastases (8%) and other organs (1%) [5,6]. In contrast, the most common site of GEP/NETs in Japanese people is the rectum, followed by the pancreas, stomach and duodenum [7,8]. Similar phenomena are seen in the Asian people including Chinese, Malay and Indian ethnic backgrounds: pancreas (38.6%), rectum (19.7%) and stomach (9.5%) [9]. GEP/NETs especially of mid-gut origin (the appendix and ileum) may secrete serotonin that causes carcinoid syndrome, and those of pancreatic islet origin may excessively secrete insulin, glucagon and gastrin. The frequency of complication of multiple endocrine neoplasia type 1 was 1%, and the frequency of symptomatic gastroenteric-NETs was 3.4% in Japan. Interestingly, 77.1% of patients with foregut-NETs had type A gastritis [7]. However, many GEP/NETs are clinically silent until late presentation of mass effects. Ito et al. [7] described there were significant differences in GEP-NETs between Japan and Western nations, primarily due to the differences in the association of non-functioning pancreatic neuroendocrine tumors in multiple endocrine neoplasia type 1 as well as their location, symptomatic status, and prevalence of malignancy.

The current WHO classification categorizes GEP/NETs into: (1) NET G1; (2) NET G2; (3) NET G3; (4) NE carcinoma (small cell or large cell) and (5) mixed NE and non-NE neoplasm [10]. At present, a total of 14 terms have been utilized to define tumors with mixed endocrine-exocrine features. Volante et al. [11] proposed a classification based on the volume of each component and structural features of the NE component. Three separate patterns can be distinguished: (1) NE tumors with focal non-NE component occupying less than 30% of the tumor, (2) mixed endocrine-exocrine carcinomas (NE or non-NE cells >30%) and (3) non-NE carcinoma with focal NE component (<30%). The type of tumor influences the prognosis: an increasing ratio of the NE component may contribute to the improvement of the prognosis [11].

The AoV is the most common location of small intestinal adenocarcinoma [12]. However, NETs involving the AoV are rare, accounting only for 0.35% of all gastrointestinal NETs. So far, 110 cases of NET of AoV origin have been reported in the literature, mostly as case reports [13]. The tumors are predominantly found on endoscopic retrograde cholangiopancreatography in patients with obstructive jaundice or acute biliary pancreatitis. The concurrence of NET with adenocarcinoma (so-called adenocarcinoid tumor) is an unusual phenomenon in the gastrointestinal tract, and has been reported in the esophagus, stomach, small intestine, vermiform appendix, colon and rectum [14-16]. In the duodenum, only three cases of adenocarcinoma synchronous with neuroendocrine neoplasm have been reported [3-5]. According to Volante et al. [11], the present case should be categorized as mixed endocrine and non-neuroendocrine neoplasm. Close clinical follow-up study for liver metastasis is needed because of lympho-vascular invasion. The expression of SSTR2, a novel marker of neuroendocrine cells [17], suggests that the tumor is susceptible to the endocrine therapy with a somatostatin analog.

It is worthy of note that not only adenocarcinoma arising from adenoma but also NET expressed CDX2, and adenocarcinoma focally contained the NE component. We suppose that NET may have occurred as a subclone of intestinal-type adenoma/adenocarcinoma component of bile duct origin.

## Conclusions

We herein described a rare case of collision tumor: adenocarcinoma of the AoV synchronous with NET, G2, of the lower part of the CBD (a mixed neuroendocrine and non-neuroendocrine carcinoma). The adenocarcinoma arose from adenoma. Not only adenoma/adenocarcinoma but also NET expressed CDX2, and adenoma/adenocarcinoma focally contained the NE component. It seems that NET has originated from intestinal-type adenoma/adenocarcinoma.
